# Uplink Sparse Channel Estimation for Hybrid Millimeter Wave Massive MIMO Systems by UTAMP-SBL

**DOI:** 10.3390/s21144760

**Published:** 2021-07-12

**Authors:** Shuai Hou, Yafeng Wang, Chao Li

**Affiliations:** School of Information and Communication Engineering, Beijing University of Posts and Telecommunications, Beijing 100876, China; houshuai@bupt.edu.cn (S.H.); lcay610@bupt.edu.cn (C.L.)

**Keywords:** millimeter-wave massive MIMO, channel estimation, sparse Bayesian learning, compressive sensing

## Abstract

The compressive sensing (CS)-based sparse channel estimator is recognized as the most effective solution to the excessive pilot overhead in massive MIMO systems. However, due to the complex signal processing in the wireless communication systems, the measurement matrix in the CS-based channel estimation is sometimes “unfriendly” to the channel recovery. To overcome this problem, in this paper, the state-of-the-art sparse Bayesian learning using approximate message passing with unitary transformation (UTAMP-SBL), which is robust to various measurement matrices, is leveraged to address the multi-user uplink channel estimation for hybrid architecture millimeter wave massive MIMO systems. Specifically, the sparsity of channels in the angular domain is exploited to reduce the pilot overhead. Simulation results demonstrate that the UTAMP-SBL is able to achieve effective performance improvement than other competitors with low pilot overhead.

## 1. Introduction

Massive MIMO is a key technology for the fifth-generation (5G) communication systems [1]. Thanks to the shorter wavelength of the millimeter wave (mmWave) signal, the numerous antennas are packed into a compact-size array, which facilitates the commercial deployment of massive MIMO systems [2], but under the consideration of the high power consumption of Analog-to-Digital Converters (ADCs), the hybrid beamforming architecture, that divides the precoder and the combiner into the analog and digital domains, is developed to solve it and regarded as an effective alternative [3,4]. In this hybrid architecture system, the precoding is crucial and dependent on the acquisition of accurate channel state information (CSI). However, the number of pilots used for channel estimation will increase linearly with that of antennas and users in the wireless systems. As a result, the channel estimation with fewer pilots is a significant challenge in the hybrid mmWave massive MIMO systems [3].

The compressive sensing (CS)-based channel estimator is capable of exploiting the sparsity of channels to reduce the pilot overhead greatly and a lot of studies on this topic have been done. Some studies focus on the sparsity in the delay domain. In [5], the orthogonal matching pursuit (OMP) algorithm is utilized to estimate the wideband mmWave delay-domain sparse channels. In [6], an adaptive structured subspace pursuit algorithm at the user is proposed to estimate the delay-domain MIMO channels with the spatio-temporal common sparsity. The authors of [7] propose a sparse channel recovery algorithm based on the vector approximate message passing (VAMP) for the massive MIMO delay-domain channels. Some works concentrate on the sparsity in the angular domain. For example, a distributed sparsity adaptive matching pursuit (DSAMP) algorithm is proposed in [8], whereby the spatially common sparsity is exploited. In [9], a novel sparse Bayesian learning (SBL) approach is utilized to recover the angular-domain block-sparse channels. In [10], a structured turbo-CS algorithm is proposed to estimate the angular-domain massive MIMO channels which are modeled by a Markov chain prior. Furthermore, the work [11] even jointly exploits the 3-D clustered structure of channels in the angular-delay domain and proposes an approximate message passing (AMP)-based estimation algorithm. However, all of above angular-domain sparse channel estimation schemes are based on the assumption that the angles exactly lie on the grids, or ignore the power leakage caused by grid mismatch. Recently, there are many off-grid channel estimation techniques. In [12], the authors utilize the low-rank structure along with the sparsity in angular domain to improve the channel estimation performance, and the off-grid angles can be recovered with their algorithm successfully. In [13], the angles are treated as random parameters and the grid-less quantized variational Bayesian channel estimation algorithm is proposed for antenna array systems with low resolution ADCs. Besides, the multi-dimensional variational line spectral estimation algorithm proposed in [14] can be effectively applied for multi-dimensional off-grid angle estimation. In addition, a novel super-resolution downlink channel estimation approach developed from the SBL is provided in [15], where the sampled angular grid points are treated as the underlying parameters.

The performance of these CS-based algorithms is affected by the measurement matrix which is usually associated with the signal processing operations in the system. If these operations are not carefully designed, the measurement matrix perhaps becomes detrimental to channel recovery. Recently, a CS algorithm, termed the SBL using approximate message passing with unitary transformation (UTAMP-SBL), is proposed by Luo et al. and it outperforms the state-of-the-art AMP-based SBL algorithms, the Gaussian generalized AMP-based SBL (GGAMP-SBL), in terms of robustness, speed and recovery accuracy for difficult measurement matrices [16]. In this paper, we apply UTAMP-SBL to the sparse channel estimation to improve the performance. Specifically, the multi-user uplink channel estimation for hybrid architecture mmWave massive MIMO systems is studied as an example. And the angular-domain sparsity of the mmWave channels is fully exploited in our estimation. Additionally, this algorithm can be extended to other sparse channel estimation for the performance improvement. Simulation results verify that the UTAMP-SBL outperforms the other competitors.

The remainder of this paper is organized as follows. In Section 2, the hybrid millimeter wave massive MIMO system model is introduced. In Section 3, the uplink sparse channel estimation with UTAMP-SBL is described. In Section 4, the Cramér-Rao bound (CRB) is provided as a performance benchmark. Simulation results are provided and the performance is discussed in Section 5. Conclusions are given in Section 6.

*Notations*: H and ⊗ denote the conjugate transpose operation and the Kronecker product. ⊙ and ⊘ represent the componentwise vector multiplication and the componentwise vector division. vec(A) denotes vectorizing the matrix A as a vector. I, 1 and 0 denote the identity matrix, the all-one column vector and the all-zero column vector, respectively. diag(a) returns a diagonal matrix with the elements of vector a on its main diagonal. $N_{C}\left(x;\mu_{x},\Sigma_{x}\right)$ represents the Gaussian distribution of the complex vector x with mean $\mu_{x}$ and covariance matrix $\Sigma_{x}$. Ga(γ;ϵ,η) denotes a Gamma distribution with shape parameter ϵ and rate parameter η. $E_{b(x)}\left[f\left(x\right)\right]$ denotes the expectation of the function f(x) with respect to probability density b(x). Finally, denotes equality up to a constant scale factor.

## 2. System Model

### 2.1. Millimeter Wave MIMO Channel Model

We consider a mmWave MIMO-OFDM system where the base station (BS) is equipped with $N_{\mathrm{BS}}$ antennas and serves *K* multi-antenna user equipments (UEs), and each UE has $N_{\mathrm{UE}}$ antennas. The frequency-domain channel between the BS and the *k*th UE at the *p*th $\left(p=1,2,\cdots ,P\right)$ subcarrier can be modeled as [17]

(1) $$H_{p,k}=\sum_{l=1}^{L}\alpha_{l,k}a_{\mathrm{BS}}\left(\theta_{l,k}\right)a_{\mathrm{UE}}^{H}\left(\phi_{l,k}\right)e^{-j2\pi f_{s}\tau_{l,k}p/P},$$

where *L* is the number of physical paths, $\alpha_{l,k}$ is the complex path gain, $f_{s}$ is the sampling rate, $\tau_{l,k}$ is the path delay, $a_{\mathrm{BS}}\left(\theta_{l,k}\right)$ and $a_{\mathrm{UE}}\left(\phi_{l,k}\right)$ are the steering vectors at the BS and the UE, respectively, $\theta_{l,k}$ and $\phi_{l,k}$ are the azimuth angles of departure and arrival (AoD/AoA) uniformly distributed in $\left[0,2\pi \right)$.

For each UE in the system, we generate one line of sight (LoS) path and L−1 non-LoS (NLoS) paths. The path gains follow the complex Gaussian distribution and the ratio of the power of LOS path to that of NLoS path is 10 dB [18]. In the OFDM system, the cyclic prefix (CP) is introduced to mitigate the inter-symbol interference and we have $\tau_{\max}f_{s}\leq N_{\mathrm{cp}}$, where $\tau_{\max}$ is the maximum path delay and $N_{\mathrm{cp}}$ is the length of CP. So we generate $\tau_{1,k}f_{s}=1$ and $\tau_{l,k}f_{s}\in \left\{2,3,\cdots ,N_{\mathrm{cp}}\right\},\;l\neq 1$. For the steering vectors, assuming the uniform linear arrays are adopted at the BS and the UE, they can be shown as

(2) $$\begin{array}{cc} \; & a_{\mathrm{BS}}\left(\theta_{l,k}\right)={\left[1,e^{j\frac{2\pi }{\lambda }d\sin\left(\theta_{l,k}\right)},\cdots ,e^{j\left(N_{\mathrm{BS}}-1\right)\frac{2\pi }{\lambda }d\sin\left(\theta_{l,k}\right)}\right]}^{T}\\ \; & a_{\mathrm{UE}}\left(\phi_{l,k}\right)={\left[1,e^{j\frac{2\pi }{\lambda }d\sin\left(\phi_{l,k}\right)},\cdots ,e^{j\left(N_{\mathrm{UE}}-1\right)\frac{2\pi }{\lambda }d\sin\left(\phi_{l,k}\right)}\right]}^{T}, \end{array}$$

where λ is the wavelength and d=λ/2 is the antenna spacing.

### 2.2. Uplink Pilot Transmission

We consider the typical hybrid analog digital precoding and combining architecture in the mmWave massive MIMO systems, where the BS and each UE are assumed to have $N_{\mathrm{BS}}^{\mathrm{RF}}$ RF chains and $N_{\mathrm{UE}}^{\mathrm{RF}}$ RF chains, respectively. We focus on the uplink pilot training. The *k*th UE transmits the pilot $s_{p,t,k}\in C^{N_{s}\times 1}$ at the *p*th pilot subcarrier and the *t*th OFDM symbol to the BS, where $N_{s}$ is the number of data streams. At the BS, the receiver receives pilots from all UEs in the same time-frequency resources and the received signal can be written as

(3) $$y_{p,t}={\left(Z_{t}^{\mathrm{RF}}Z_{p,t}^{\mathrm{BB}}\right)}^{H}\left(\sum_{k=1}^{K}H_{p,k}F_{t,k}^{\mathrm{RF}}F_{p,t,k}^{\mathrm{BB}}s_{p,t,k}+n_{p,t}\right),$$

where $F_{p,t,k}^{\mathrm{BB}}\in C^{N_{\mathrm{UE}}^{\mathrm{RF}}\times N_{s}}$ and $F_{t,k}^{\mathrm{RF}}\in C^{N_{\mathrm{UE}}\times N_{\mathrm{UE}}^{\mathrm{RF}}}$ are the digital precoder and the analog precoder of the *k*th user, $Z_{p,t}^{\mathrm{BB}}\in C^{N_{\mathrm{BS}}^{\mathrm{RF}}\times N_{s}}$ and $Z_{t}^{\mathrm{RF}}\in C^{N_{\mathrm{BS}}\times N_{\mathrm{BS}}^{\mathrm{RF}}}$ are the digital combiner and the analog combiner of the BS and $n_{p,t}\in C^{N_{\mathrm{BS}}\times 1}$ is the additive white Gaussian noise (AWGN). Note that the analog precoder and the analog combiner are frequency-flat, while the digital precoder and the digital combiner are different for each subcarrier. This is because the RF phase shifters can provide constant response over the wide frequency band when the fully connected network is used.

### 2.3. Sparse Channel Estimation Formulation

In the multi-user uplink channel estimation, the channels of *K* users, $H_{p,k}\in C^{N_{\mathrm{BS}}\times N_{\mathrm{UE}}}$, k=1,2,⋯,K, need to be estimated from the received signal $y_{p,t}$. It is clear that there are $KN_{\mathrm{BS}}N_{\mathrm{UE}}$ non-zero elements in these *K* channel matrices. Because there are only $N_{s}$ elements in $y_{p,t}$, we have to arrange $KN_{\mathrm{BS}}N_{\mathrm{UE}}/N_{s}$ pilots for channel estimation and the pilot overhead is unacceptable. Fortunately, we benefit from the inherent sparse characteristic of millimeter wave channels and apply the CS algorithms in the channel estimation to reduce the pilot overhead.

**Figure 1:**
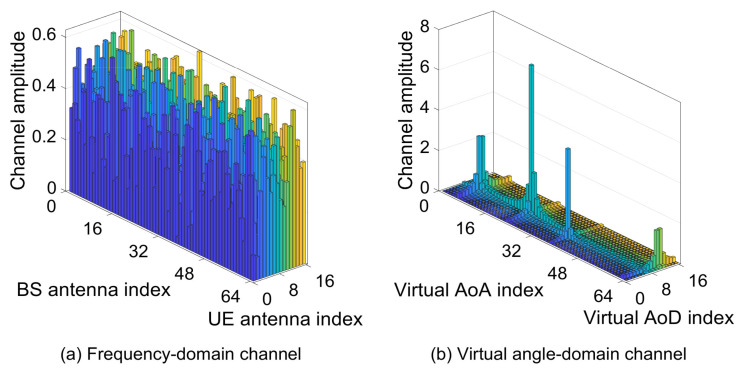
The illustration of the frequency-domain channel and the virtual angle-domain channel.

In order to formulate the sparse channel estimation problem, we first rewrite the frequency-domain channel matrix $H_{p,k}\in C^{N_{\mathrm{BS}}\times N_{\mathrm{UE}}}$ as

(4) $$H_{p,k}=A_{\mathrm{BS}}H_{p,k}^{\omega }A_{\mathrm{UE}}^{H},$$

where $H_{p,k}^{\omega }\in C^{G_{\mathrm{BS}}\times G_{\mathrm{UE}}}$ is the virtual angle-domain channel matrix, $A_{\mathrm{BS}}\in C^{N_{\mathrm{BS}}\times G_{\mathrm{BS}}}$ and $A_{\mathrm{UE}}\in C^{N_{\mathrm{UE}}\times G_{\mathrm{UE}}}$ are the partial discrete Fourier transform (DFT) matrices, $G_{\mathrm{BS}}$ and $G_{\mathrm{UE}}$ are the numbers of grids at the BS and the UE. Because of the domination of the LOS path in the multipath channel, the virtual angle-domain mmWave channel appears the sparsity, i.e., there are only a few non-zero elements in the channel matrix $H_{p,k}^{\omega }$ and other components are zero. Actually, these zeros are not strictly equal to zero but are only close to zero owing to the power leakage problem [19], which is ignored in this paper for simplification and it will be disscussed in the future research. The frequency-domain channel $H_{p,k}$ and the transformed virtual angle-domain channel $H_{p,k}^{\omega }$ are shown in Figure 1, where the frequency-domain channel is generated according to (1) and the virtual angle-domain channel is derived from (4). Obviously, from Figure 1b, the power of the channel is concentrated in a few virtual angles and these channel components can be estimated from the low-overhead pilot.

Substituting (4) in (3), we have

(5) $$\begin{array}{c} y_{p,t}=Z_{p,t}^{H}\sum_{k=1}^{K}A_{\mathrm{BS}}H_{p,k}^{\omega }A_{\mathrm{UE}}^{H}x_{p,t,k}+{\bar{n}}_{p,t}, \end{array}$$

where $Z_{p,t}=Z_{t}^{\mathrm{RF}}Z_{p,t}^{\mathrm{BB}}\in C^{N_{\mathrm{BS}}\times N_{s}}$ is the combiner at the BS, $x_{p,t,k}=F_{t,k}^{\mathrm{RF}}F_{p,t,k}^{\mathrm{BB}}s_{p,t,k}\in C^{N_{\mathrm{UE}}\times 1}$ is the pilot after precoding at the UE and ${\bar{n}}_{p,t}=Z_{p,t}^{H}n_{p,t}\in C^{N_{s}\times 1}$ is the noise after combining at the BS. Note that usually the channel $H_{p,k}^{\omega }$ is invariant in some successive OFDM symbols. Rewrite (5) in matrix form as

(6) $$y_{p,t}=Z_{p,t}^{H}A_{\mathrm{BS}}{\bar{H}}_{p}^{\omega }{\bar{A}}_{\mathrm{UE}}^{H}{\bar{x}}_{p,t}+{\bar{n}}_{p,t},$$

where ${\bar{H}}_{p}^{\omega }=\left[H_{p,1}^{\omega },H_{p,2}^{\omega },\cdots ,H_{p,K}^{\omega }\right]\in C^{G_{\mathrm{BS}}\times KG_{\mathrm{UE}}}$, ${\bar{A}}_{\mathrm{UE}}^{H}=\mathrm{diag}\left(A_{\mathrm{UE}}^{H},A_{\mathrm{UE}}^{H},\cdots ,A_{\mathrm{UE}}^{H}\right)\in C^{KG_{\mathrm{UE}}\times KN_{\mathrm{UE}}}$, and ${\bar{x}}_{p,t}={\left[x_{p,t,1}^{T},x_{p,t,2}^{T},\cdots ,x_{p,t,K}^{T}\right]}^{T}\in C^{KN_{\mathrm{UE}}\times 1}$. Using the result $\mathrm{vec}\left(ABC\right)$$=\left(C^{T}⊗A\right)\mathrm{vec}\left(B\right)$, (6) can be equivalently expressed as

(7) $$\begin{array}{cc} y_{p,t} & =\mathrm{vec}\left(Z_{p,t}^{H}A_{\mathrm{BS}}{\bar{H}}_{p}^{\omega }{\bar{A}}_{\mathrm{UE}}^{H}{\bar{x}}_{p,t}\right)+{\bar{n}}_{p,t}\\ \; & =\left({\left({\bar{A}}_{\mathrm{UE}}^{H}{\bar{x}}_{p,t}\right)}^{T}⊗\left(Z_{p,t}^{H}A_{\mathrm{BS}}\right)\right)\mathrm{vec}\left({\bar{H}}_{p}^{\omega }\right)+{\bar{n}}_{p,t}\\ \; & =\Phi_{p,t}h_{p}^{\omega }+{\bar{n}}_{p,t}, \end{array}$$

where $\Phi_{p,t}=\left({\left({\bar{A}}_{\mathrm{UE}}^{H}{\bar{x}}_{p,t}\right)}^{T}⊗\left(Z_{p,t}^{H}A_{\mathrm{BS}}\right)\right)\in C^{N_{s}\times KG_{\mathrm{BS}}G_{\mathrm{UE}}}$, $h_{p}^{\omega }=\mathrm{vec}\left({\bar{H}}_{p}^{\omega }\right)\in C^{KG_{\mathrm{BS}}G_{\mathrm{UE}}\times 1}$. We stack the received signals that are transmited on *G* successive symbols, and we have

(8) $$y_{p}=\Phi_{p}h_{p}^{\omega }+{\bar{n}}_{p},$$

where $y_{p}={\left[y_{p,1}^{T},y_{p,2}^{T},\cdots ,y_{p,G}^{T}\right]}^{T}\in C^{GN_{s}\times 1}$, $\Phi_{p}={\left[\Phi_{p,1}^{T},\Phi_{p,2}^{T},\cdots ,\Phi_{p,G}^{T}\right]}^{T}\in C^{GN_{s}\times KG_{\mathrm{BS}}G_{\mathrm{UE}}}$ and ${\bar{n}}_{p}={\left[n_{p,1}^{T},n_{p,2}^{T},\cdots ,n_{p,G}^{T}\right]}^{T}\in C^{GN_{s}\times 1}$.

As shown above, in the channel estimation, the measurement matrix $\Phi_{p}$ derives from pilots, precoders, combiners and the partial DFT matrices, so it is a difficult matrix in all probability. With the difficult measurement matrix, the existing sparse channel estimation algorithms can fail to recover channels. As a result, the UTAMP-SBL which is robust to difficult measurement matrix is a wise choice.

## 3. Uplink Channel Estimation with UTAMP-SBL

Focus on the channel estimation of the single subcarrier and the subscript *p* is omitted for conciseness,

(9) $$y=\Phi h^{\omega }+\bar{n}.$$

Let $M=GN_{s}$, $N=KG_{\mathrm{BS}}G_{\mathrm{UE}}$, so the sizes of y, Φ and $h^{\omega }$ are M×1, M×N and N×1, respectively, where M≪N. In order to characterize the sparsity of $h^{\omega }$, the Gaussian prior model is adopted, i.e.,

(10) $$p\left(h^{\omega }\right)=\int p_{h^{\omega }}\left(h^{\omega }|\gamma \right)p_{\gamma }\left(\gamma \right)\;d\gamma ,$$

where $p_{h^{\omega }}\left(h^{\omega }|\gamma \right)=N_{C}\left(h^{\omega };0,\mathrm{diag}\left(\gamma \right)\right)$ and $p_{\gamma }\left(\gamma \right)=\mathrm{Ga}\left(\gamma ;\epsilon ,\eta \right)$. Define the singular value decomposition (SVD) of Φ is Φ=UΣV, where U is an orthogonal matrix and $U^{H}U=I$. Perform unitary transformation to (9) and we have

(11) $$\begin{array}{cc} U^{H}y & =U^{H}\Phi h^{\omega }+U^{H}\bar{n}\\ r & =\Sigma Vh^{\omega }+w, \end{array}$$

where $r=U^{H}y\in C^{M\times 1}$, $w=U^{H}\bar{n}\in C^{M\times 1}$ and w follows $N_{C}\left(w;0,\sigma I\right)$. Define $z=\Sigma Vh^{\omega }$ and the likelihood function of z is

(12) $$p_{r}\left(r|z;\sigma \right)=N_{C}\left(r;z,\sigma I\right).$$

**Figure 2:**
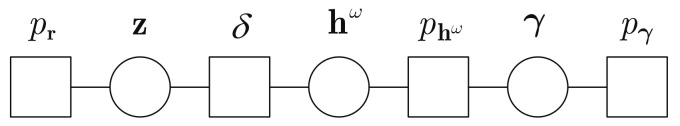
The factor graph of uplink sparse channel estimation.

According to the Bayesian rule, the joint probability density function is

(13) $$\begin{array}{c} p\left(r,z,h^{\omega };\sigma ,\epsilon ,\eta \right)=p_{r}\left(r|z;\sigma \right)\delta \left(z-\Sigma Vh^{\omega }\right)p\left(h^{\omega }\right), \end{array}$$

where the Dirac function factor δ(·) is adopted first in [20] to facilitate the derivation of the algorithm. The factor graph of the factorization (13) is shown in Figure 2. The UTAMP-SBL algorithm will be derived from message-passing on this graph and applied to the channel estimation.

Firstly, we consider the massages between node $p_{r}$ and node z. According to the sum-product algorithm, the massage from function node $p_{r}$ to variable node z is $m_{p_{r}\to z}\left(z\right)=p_{r}\left(r|z;\sigma \right)=N_{C}\left(z;r,\hat{\sigma }I\right)$. The massage from variable node z to function node $p_{r}$ is defined as $m_{z\to p_{r}}\left(z\right)=N_{C}\left(z;\mu ,\mathrm{diag}(\tau_{\mu })\right)$. So the belief $b\left(z\right)=m_{z\to p_{r}}\left(z\right)m_{p_{r}\to z}\left(z\right)$ can be calculated as $b\left(z\right)=N_{C}\left(z;\hat{z},\mathrm{diag}\left(v_{z}\right)\right)$, where the mean and variance are

(14) $$\begin{array}{cc} v_{z} & =1⊘\left(1⊘\tau_{\mu }+\frac{1}{\hat{\sigma }}1\right),\\ \hat{z} & =v_{z}⊙\left(\frac{r}{\hat{\sigma }}+\mu ⊘\tau_{\mu }\right). \end{array}$$

Then, we focus on the massages between node $p_{h^{\omega }}$ and node $h^{\omega }$. Similarly, the massage from function node $p_{h^{\omega }}$ to variable node $h^{\omega }$ is defined as $m_{p_{h^{\omega }}\to h^{\omega }}\left(h^{\omega }\right)=N_{C}\left(h^{\omega };0,\mathrm{diag}\left(\hat{\gamma }\right)\right)$. And the massage from variable node $h^{\omega }$ to function node $p_{h^{\omega }}$ is defined as $m_{h^{\omega }\to p_{h^{\omega }}}\left(h^{\omega }\right)=N_{C}\left(h^{\omega };\pi ,\tau_{\pi }I\right)$. So the belief $b\left(h^{\omega }\right)=m_{h^{\omega }\to p_{h^{\omega }}}\left(h^{\omega }\right)m_{p_{h^{\omega }}\to h^{\omega }}\left(h^{\omega }\right)$ is calculated as $b\left(h^{\omega }\right)=N_{C}\left(h^{\omega };{\hat{h}}^{\omega },v_{h^{\omega }}I\right)$, where the mean and variance are

(15) $$\begin{array}{cc} v_{h^{\omega }} & =\frac{1}{N}1^{T}\left(1⊘\left(1/\tau_{\pi }+1⊘\hat{\gamma }\right)\right),\\ {\hat{h}}^{\omega } & =\pi ⊘\left(1+\tau_{\pi }1⊘\hat{\gamma }\right). \end{array}$$

For the sparse channel estimation, the mean of the belief $b(h^{\omega })$ is the estimated channel.

Next, the variable μ, $\tau_{\mu }$, π and $\tau_{\pi }$ mentioned above can be updated with the UTAMP which is derived from the AMP. In the UTAMP, the means and variances are calculated by [16]

(16) $$\begin{array}{cc} \tau_{\mu } & =v_{h^{\omega }}\Sigma \Sigma^{H}1,\\ \mu & =\Sigma V{\hat{h}}^{\omega }-\tau_{\mu }⊙s,\\ \tau_{\pi } & ={\left(\frac{1}{N}\left({\left(\Sigma \Sigma^{H}1\right)}^{H}\tau_{s}\right)\right)}^{-1},\\ \pi & ={\hat{h}}^{\omega }+\tau_{\pi }\left({\left(\Sigma V\right)}^{H}s\right), \end{array}$$

where the intermediate variables s and $\tau_{s}$ are given by [16]

(17) $$\begin{array}{cc} \tau_{s} & =1⊘\left(\tau_{\mu }+\hat{\sigma }1\right),\\ s & =\tau_{s}⊙\left(r-\mu \right). \end{array}$$

Besides, there are several model parameters need to be learned at each iteration. For the noise precision $\hat{\sigma }$, it can be updated with the expectation-maximization (EM) algorithm [16]

(18) $$\hat{\sigma }=\frac{1}{M}\left(∥r-\hat{z}{∥}^{2}+1^{T}v_{z}\right).$$

For the variable γ, according to the sum-product algorithm, the componentwise massage from function node $p_{h_{n}^{\omega }}$ to variable node $\gamma_{n}$ is

(19) $$\begin{array}{cc} m_{p_{h_{n}^{\omega }}\to \gamma_{n}}\left(\gamma_{n}\right) & =p_{h_{n}^{\omega }}\left(\gamma_{n}\right)m_{h_{n}^{\omega }\to p_{h_{n}^{\omega }}}\left(\gamma_{n}\right)\\ \; & =N_{C}\left(h_{n}^{\omega };0,\gamma_{n}\right)N_{C}\left(h_{n}^{\omega };\pi_{n},\tau_{\pi }\right)\\ \; & \sqrt{\frac{1}{\gamma_{n}}}\exp\left\{-\frac{1}{2\gamma_{n}}\left({\left|{\hat{h}}_{n}^{\omega }\right|}^{2}+v_{h^{\omega }}\right)\right\}. \end{array}$$

The message from variable node γ to function node $p_{h^{\omega }}$ is $m_{\gamma \to p_{h^{\omega }}}\left(\gamma \right)=m_{p_{\gamma }\to \gamma }\left(\gamma \right)=\mathrm{Ga}\left(\gamma ;\epsilon ,\eta \right)$. Consequently, the belief $b(\gamma_{n})$ is expressed as

(20) $$\begin{array}{cc} b(\gamma_{n}) & =m_{\gamma_{n}\to p_{h_{n}^{\omega }}}\left(\gamma_{n}\right)m_{p_{h_{n}^{\omega }}\to \gamma_{n}}\left(\gamma_{n}\right)\\ \; & \gamma_{n}^{\frac{1}{2}-\frac{1}{2}\hat{\epsilon }}\exp\left\{-\frac{1}{2\gamma_{n}}\left({\left|{\hat{h}}_{n}^{\omega }\right|}^{2}+v_{h^{\omega }}+2\eta \right)\right\}. \end{array}$$

So, ${\hat{\gamma }}_{n}$ is the mean of this distribution, i.e.,

(21) $${\hat{\gamma }}_{n}=\frac{{\left|{\hat{h}}_{n}^{\omega }\right|}^{2}+v_{h^{\omega }}}{\hat{\epsilon }+1},$$

when we set η=0. For the parameter $\hat{\epsilon }$, it is difficult to renewed, but there is an effective way found in [16] and we use it directly. As a result, $\hat{\epsilon }$ is calculated by

(22) $$\hat{\epsilon }=\frac{1}{2}\sqrt{\log\left(\frac{1}{N}\sum_{n}\frac{1}{{\hat{\gamma }}_{n}}\right)-\frac{1}{N}\sum_{n}\log\frac{1}{{\hat{\gamma }}_{n}}}.$$

The process of the channel estimation with UTAMP-SBL is given in Algorithm 1. In this algorithm, the iteration will be halted if the number of it reaches the maximum value $T_{\max}$ or $∥{\hat{h}}^{\omega (t)}-{\hat{h}}^{\omega (t-1)}∥/∥{\hat{h}}^{\omega (t)}∥<10^{-12}$.
**Algorithm 1** Uplink channel estimation with UTAMP-SBL.**Input:** The received signal y and the matrix Φ. **Output:** The estimated sparse channel ${\hat{h}}^{\omega }$.
1:**Initialization**: SVD Φ=UΣV, $r=U^{H}y$, $v_{h^{\omega }}=1$, ${\hat{h}}^{\omega }=0$, s=0, $\hat{\sigma }=1$, $\hat{\gamma }=1$, $\hat{\epsilon }=0.001$2:**repeat**3: Calculate the mean μ and variance $\tau_{\mu }$ as (16).4: Calculate the mean $\hat{z}$ and variance $v_{z}$ as (14).5: Update the noise variance $\hat{\sigma }$ as (18).6: Calculate the mean s and variance $\tau_{s}$ as (17).7: Calculate the mean π and variance $\tau_{\pi }$ as (16).8: Calculate the mean ${\hat{h}}^{\omega }$ and variance $v_{h^{\omega }}$ as (15).9: Calculate the variance $\hat{\gamma }$ according to (21).10: Update the parameter $\hat{\epsilon }$ according to (22).11:**until halt**


The computational complexity of the UTAMP-SBL is dominated by the complex multiplications required for matrix-vector operations. At the stage of initialization, the complexity of SVD is $O(M^{2}N)$ [21]. The matrix-vector product of $U^{H}y$ is $O(M^{2})$. At the stage of iteration, the complexity of calculating $\tau_{\mu }$ and μ are respectively $O(M^{2}+2M)$ and O(2MN+M) in the step 3 of Algorithm 1. The calculations of remaining steps only involve the component-wise vector multiplication or scalar operations which bring a small amount of complex multiplication compared with matrix-vector product. Therefore, the computational complexity of them is omitted. When the iteration reaches *T*, the total complexity is $O(M^{2}N+M^{2}+T\left(M^{2}+2M+2MN+M\right))$. Discarding the low-order terms, the total complexity is $O(M^{2}N)$.

## 4. Cramér-Rao Bound Analysis

In this section, the CRB is provided as a performance benchmark of the channel estimation. From Section 3, the sparse channel estimation is modeled as an SBL problem and the CRBs for SBL derived in [22] can be adopted directly.

According to [22], the lower bound on the mean squared error (MSE) matrix of $h^{\omega }$ is

(23) $$E⪰{\left(\frac{\Phi^{H}\Phi }{{\hat{\sigma }}^{2}}+\mathrm{diag}\left(\hat{\gamma }\right)\right)}^{-1},$$

where $\hat{\sigma }$ is from (18) and the *n*th entry of $\hat{\gamma }={\left[{\hat{\gamma }}_{1},{\hat{\gamma }}_{2},\cdots ,{\hat{\gamma }}_{N}\right]}^{T}$ is gotten from (21). Thus, the CRB of ${\hat{h}}^{\omega }$ is

(24) $$\mathrm{CRB}\left({\hat{h}}^{\omega }\right)=\frac{1}{N}\mathrm{tr}\left(E\right).$$

## 5. Simulation Results

The simulation parameters are chosen as follows, $N_{\mathrm{BS}}=G_{\mathrm{BS}}=64$, $N_{\mathrm{UE}}=G_{\mathrm{UE}}=16$, $N_{\mathrm{BS}}^{\mathrm{RF}}=N_{\mathrm{UE}}^{\mathrm{RF}}=4$, $N_{s}=4$, K=4, P=32 and L=4. The pilots, precoders and combiners are generated according to [17]. In order to evaluate the performance, the normalized MSE (NMSE) $E\left\{\frac{∥h^{\omega }-{\hat{h}}^{\omega }{∥}^{2}}{∥h^{\omega }{∥}^{2}}\right\}$ between the estimated channel ${\hat{h}}^{\omega }$ and the real channel $h^{\omega }$ is introduced. We compare the NMSE performance for the OMP [5], SBL [9], EM-BBG-VAMP [7], SuRe-CSBL [15], TurboCS [10], UTAMP-SBL, and the CRB derived in Section 4.

**Figure 3:**
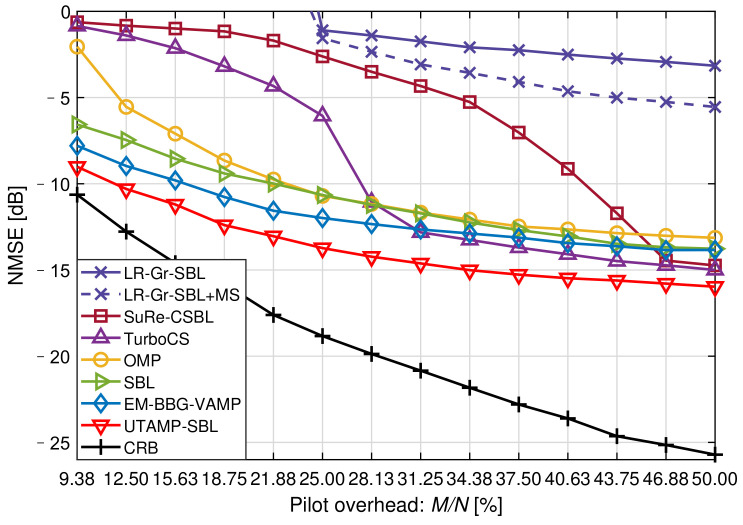
The NMSE versus the pilot overhead at SNR = 20 dB.

Figure 3 shows the NMSE versus the pilot overhead at SNR=20dB. As defined in Section 3, *N* is the dimension of the received signal y and *M* is the dimension of the sparse channel $h^{\omega }$. In this simulation, the pilot overhead is denoted as M/N. We can find that the off-grid algorithm, SuRe-CSBL, could not achieve the desired performance when the pilot is insufficient in spite of it is the winners in [15]. The SBL, EM-BBG-VAMP and UTAMP-SBL work well with 9.38% pilot overhead compared with other algorithms. Thus, when the pilot overhead is extremely low, the SuRe-CSBL, TurboCS and OMP are not sensible choices. It is also shown that the UTAMP-SBL performs distinctly better than the other estimators with the same pilot overhead. From another side, it is also concluded that the UTAMP-SBL can accurately estimate the channels with fewer pilots. For example, when the NMSE performance meets −10 dB, the OMP, SBL and EM-BBG-VAMP respectively need 22.66%, 21.88%, 16.41% pilot overhead. However, the UTAMP-SBL only needs 11.72% overhead, which means the pilot overhead is reduced by 28.58% compared with the EM-BBG-VAMP.

**Figure 4:**
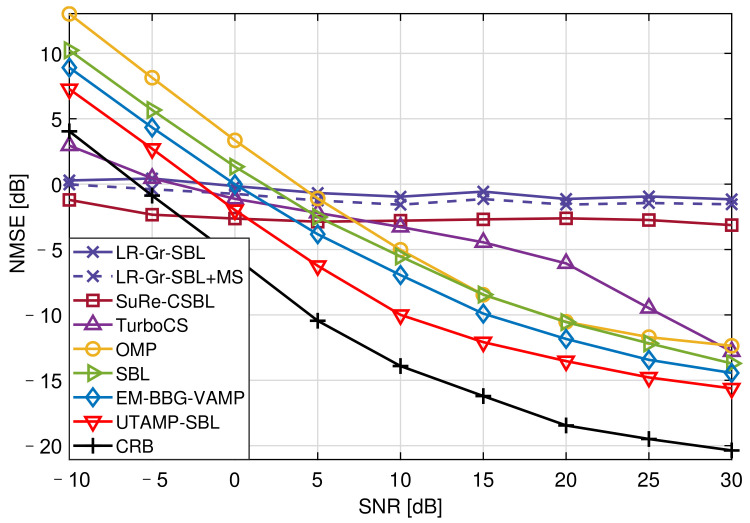
The NMSE versus the SNR with 25.00% pilot overhead.

Figure 4 shows the NMSE versus the SNR with 25.00% pilot overhead. Due to insufficient of pilots, the SuRe-CSBL and TurboCS could not estimate the channels well, even if their NMSEs are lower than CRB at low SNRs. It is observed that the UTAMP-SBL has the best performance compared with the other competitors. This is because the UTAMP-SBL is robust to different types of difficult measurement matrices, such as non-zero mean, rank-deficient, correlated, or ill-conditioned matrix [16]. This feature is crucial for the practical application of this algorithm. In the channel estimation, the measurement matrix $\Phi_{p}$ is usually related to the complex signal processing operations as described in Section 2.3, so it can be a difficult matrix. Therefore, the UTAMP-SBL with strong robustness performs better in the channel estimation. Moreover, the matrix inversion step of SBL causing the high complexity is avoided by replacing the E-step in the EM with UTAMP, and the algorithm complexity is reduced [16].

**Figure 5:**
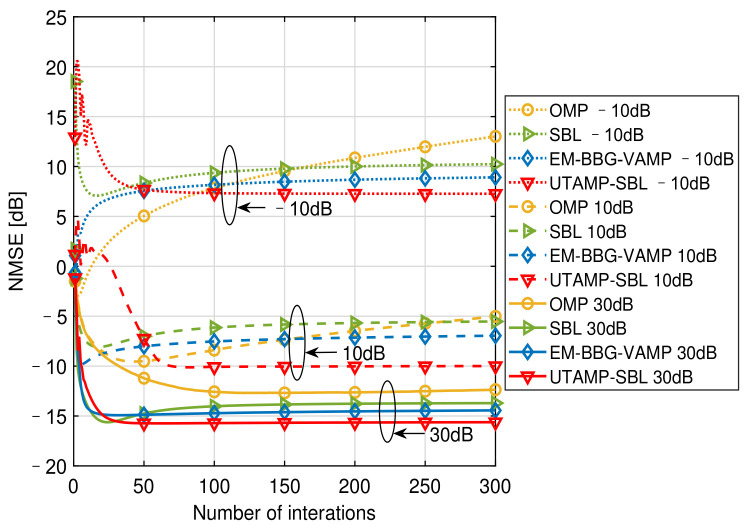
The NMSE versus the number of iterations at different SNRs with 25.00% pilot overhead.

Figure 5 provides the NMSE versus the number of iterations at different SNRs with 25.00% pilot overhead, which illustrates the convergences of the algorithms. Since the algorithms, SuRe-CSBL, TurboCS, are not suitable for our channel estimation when the pilot overhead is 25.00%, only the convergences of the OMP, SBL, EM-BBG-VAMP and UTAMP-SBL are compared. When SNR=−10dB, the OMP fails to converge even if the number of iterations reaches 300, and the NMSE performance decreases with the increasing iterations. This shows that the OMP is not suitable for the low SNR cases. The SBL, EM-BBG-VAMP and UTAMP-SBL require about 100 iterations to converge. When SNR=10dB, the OMP is still with the worst convergence and the UTAMP-SBL is with the best convergence. However, when SNR=30dB, the EM-BBG-VAMP converges slightly faster than the UTAMP-SBL. From the results, the EM-BBG-VAMP converges within 20 iterations while the UTAMP-SBL requires about 40 iterations. And these two algorithms are both faster than the SBL and the OMP. Additionally, in this figure, only the performance of the UTAMP-SBL becomes better with the iteration at all SNRs, while that of other algorithms sometimes deteriorates with the iteration. This further demonstrates the advantage of the UTAMP-SBL in convergence. From above observation and analysis, the UTAMP-SBL is superior to the comparison algorithms in the convergence speed and the NMSE performance.

## 6. Conclusions

In this paper, the UTAMP-SBL algorithm is applied to estimate the sparse channels for improving the performance. In order to evaluate the effect of this algorithm in practical channel estimation, we study the multi-user uplink channel estimation for hybrid architecture mmWave massive MIMO systems. Simulation results demonstrate the superiority of the UTAMP-SBL in terms of NMSE performance and pilot overhead reduction.

## Data Availability

Not applicable.
